# Establishing and operating a ‘virtual ward’ system to provide care for patients with COVID-19 at home: experience from The Gambia

**DOI:** 10.1136/bmjgh-2021-005883

**Published:** 2021-06-17

**Authors:** Oghenebrume Wariri, Uduak Okomo, Carla Cerami, Emmanuel Okoh, Francis Oko, Hawanatu Jah, Kalifa Bojang, Bubacarr Susso, Yekini Olatunji, Esin Nkereuwem, Fatai Momodou Akemokwe, Modou Jobe, Orighomisan Freda Agboghoroma, Bunja Kebbeh, Ghata Sowe, Thomas Gilleh, Naffie Jobe, Effua Usuf, Ed Clarke, Helen Brotherton, Karen Forrest

**Affiliations:** 1 Vaccines and Immunity Theme, Medical Research Council Unit, The Gambia at London School of Hygiene and Tropical Medicine, Banjul, The Gambia; 2 Nutrition Theme, Medical Research Council Unit, The Gambia at London School of Hygiene and Tropical Medicine, Banjul, The Gambia; 3 Disease Control and Elimination, Medical Research Council Unit The Gambia, at London School of Hygiene and Tropical Medicine, Banjul, Gambia; 4 Clinical Services Department, Medical Research Council Unit The Gambia, at London School of Hygiene and Tropical Medicine, Banjul, Gambia; 5 Data Management and Archives, Medical Research Council Unit The Gambia, at London School of Hygiene and Tropical Medicine, Banjul, Gambia; 6 Directorate, Medical Research Council Unit The Gambia, at London School of Hygiene and Tropical Medicine, Banjul, Gambia; 7 Infectious Disease Epidemiology, London School of Hygiene and Tropical Medicine, London, UK

**Keywords:** COVID-19, control strategies, health systems

## Abstract

Health systems in sub-Saharan Africa have remained overstretched from dealing with endemic diseases, which limit their capacity to absorb additional stress from new and emerging infectious diseases. Against this backdrop, the rapidly evolving COVID-19 pandemic presented an additional challenge of insufficient hospital beds and human resource for health needed to deliver hospital-based COVID-19 care. Emerging evidence from high-income countries suggests that a ‘virtual ward’ (VW) system can provide adequate home-based care for selected patients with COVID-19, thereby reducing the need for admissions and mitigate additional stress on hospital beds. We established a VW at the Medical Research Council Unit, The Gambia at the London School of Hygiene and Tropical Medicine, a biomedical research institution located in The Gambia, a low-income west African country, to care for members of staff and their families infected with COVID-19. In this practice paper, we share our experience focusing on the key components of the system, how it was set up and successfully operated to support patients with COVID-19 in non-hospital settings. We describe the composition of the multidisciplinary team operating the VW, how we developed clinical standard operating procedures, how clinical oversight is provided and the use of teleconsultation and data capture systems to successfully drive the process. We demonstrate that using a VW to provide an additional level of support for patients with COVID-19 at home is feasible in a low-income country in sub-Saharan Africa. We believe that other low-income or resource-constrained settings can adopt and contextualise the processes described in this practice paper to provide additional support for patients with COVID-19 in non-hospital settings.

**Summary box**

- The ‘virtual ward’ (VW) system for supporting patients with COVID-19 has been successfully implemented in some high-income countries and has been shown to reduce admissions and mitigate pressure on hospital beds.
- We demonstrate that a VW to support patients with COVID-19 at home is feasible in a low-income country in sub-Saharan Africa.
- Other low-income or resource-constrained settings can adopt and contextualise the processes we have described in this practice paper to provide an additional level of support for patients with COVID-19 in non-hospital settings who are usually left with no clinical support.

## Introduction

From the time it was first confirmed in December 2019, COVID-19 has spread to six continents, with more than 119 million cases and 2.6 million deaths registered as of mid-March 2021.^1^ The first case of COVID-19 in Africa was confirmed on 14 February 2020 in Egypt and by mid-April 2020, there were already 19 895 cases reported across 52 of the 54 countries in the continent.^2^ As with other pandemics, the COVID-19 pandemic has had direct and indirect effects on populations in every country.^3^ While the reported effects on morbidity and mortality appear to be less intense in some countries,^4^ the staggering levels of morbidity and mortality in other countries together with the increased need for facility care have severely stretched, in some cases overwhelmed, their health systems—irrespective of the per capital gross national income.^5^

Health systems in Africa were already stretched thin from dealing with endemic infectious disease, non-communicable diseases, maternal and child health issues prior to the onset of the COVID-19 pandemic.^6^ In this context, their capacity to absorb additional stress from a widespread outbreak was limited. Early mathematical models had suggested that Africa could be the most vulnerable continent.^7 8^ It was also anticipated that higher incidence of severe forms of COVID-19 could occur in Africa due to the high prevalence of conditions that impair the immune system: tuberculosis, HIV/AIDS, malaria, anaemia and underlying malnutrition.^9^ Furthermore, there was the additional challenge of insufficient critical care beds and human resources for health (HRH) needed to support severe forms of COVID-19. This led to shifts of HRH from other areas to support COVID-19 care, thus creating shortages of clinical staff to support routine health services.^10^ The shortages of HRH needed to sustain routine services have dire consequences such as the reduction in maternal, newborn and child health services, which might halt progress towards global health targets.^11^

As the pandemic progressed, there was a reorganisation of the health service delivery systems to mitigate the potential impact of the crisis. While there is a global consensus regarding the general principles of prevention and case management, many countries have also implemented context specific strategies that suited their local realities,^12^ such as the adoption of digital communication tools to deliver safe virtual care for COVID-19 patients and to coordinate the response to the pandemic.^13^ Digital health or telemedicine holds enormous promise as it reduces the risk of exposure to COVID-19 by health workers, takes the pressure off the healthcare system and keeps clinical staff and patients connected even when they must be physically apart.

Remote home monitoring of patients also known as the ‘virtual ward’ (VW) system involves the use of information and communications technology platforms by clinical teams to provide care for patients.^14^ VW systems have been used to provide care for patients with other medical conditions prior to the onset of COVID-19, with varied implementation models and patient outcomes.^15^ Since the onset of the COVID-19 pandemic, reported VW systems have used digital platforms,^16 17^ and/or paper-based diaries with accompanying telephone calls^18–20^ to care for patients. The majority of COVID-19 VW systems reported so far have been implemented in the USA and UK^21^ and have been shown to reduce the need for admissions and mitigate pressure on hospital beds. However, the focus of existing literature on COVID-19 VW systems has been the clinical outcomes associated with the system, with less on the experience and processes involved in setting up and operationalising such systems.^22^ To the best of our knowledge, there is no literature describing the use of the VW system to support patients with COVID-19 in sub-Saharan African countries.^15^ In this practice paper, we hope to bridge this important gap by sharing our experience of setting up and operating a VW system to provide care for patients with COVID-19 in a biomedical research institution in The Gambia, West Africa.

## Setting and context

**Figure 1:**
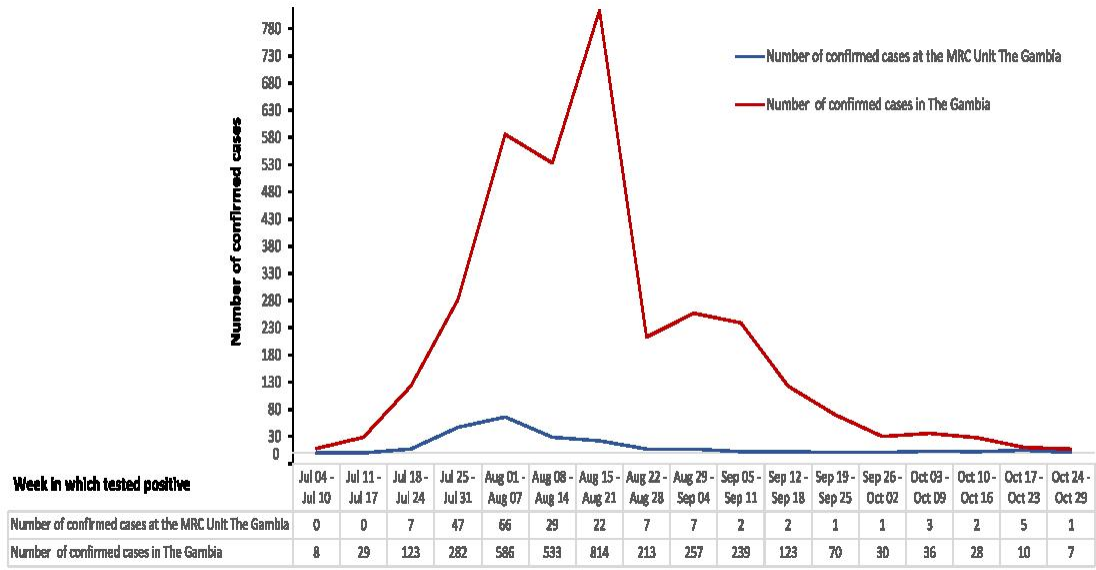
Weekly Epi-curve of confirmed COVID-19 cases among MRCG at LSHTM staff and the national (The Gambia) cases from 4 July 2020 to 29 October 2020 (first wave of COVID-19 in The Gambia). Sources: MRCG at LSHTM cases (internal database) and total cases in The Gambia (www.moh.gov.gm). LSHTM, London School of Hygiene and Tropical Medicine; MRCG, Medical Research Council Unit The Gambia.

The Gambia is a low-income west African country, with a per capita health expenditure in 2017 of US$23,^23^ and has a population of 2.4 million people.^24^ There are estimated 11 hospital beds per 10 000 of the population and one physician per 10 000 of the population as of 2015.^25^ The first case of COVID-19 in The Gambia was confirmed on 17 March 2020 as an imported case in an adult female. By the end of June 2020, just over 3 months since the index case was confirmed, only 48 additional cases were detected in the country. However, between July 2020 and September 2020, the number of cases rapidly increased and peaked, with 3579 COVID-19 cases (ie, 15 cases per 10 000 population) confirmed by the end of September 2020 (figure 1).

Until late August 2020, the national case management policy specified that all confirmed COVID-19 cases be isolated in government designated treatment centres where they would be managed until they tested negative twice before discharge.^26^ Individuals who were considered to have been significant contacts of positive cases were also quarantined in designated hotels at no personal cost. As the number of confirmed cases peaked, there was a significant strain on hospital beds at the designated national treatment centres. This situation was due, in part, to the national criteria that required in-facility care for all confirmed cases and two negative SARS-CoV-2 PCR test before their discharge from in-hospital care. There was, however, a national change to the WHO symptomatic criteria for releasing patients with COVID-19 from isolation in late August 2020.^27^

The Medical Research Council Unit The Gambia at London School of Hygiene and Tropical Medicine (MRCG at LSHTM) is a biomedical research institution operational in The Gambia for more than 70 years and has over 1300 employees. The MRCG at LSHTM has a 42-bed Clinical Services Department (CSD), which provides outpatient and inpatient clinical care for its staff and their relatives, study participants and the local population. Apart from these responsibilities, the CSD is one of only two hospital facilities in The Gambia that supports inpatient management of patients with severe COVID-19. As part of a wider COVID-19 preparedness, at the beginning of the pandemic, the MRCG at LSHTM made several changes to its clinical systems in the CSD such as setting up an additional 30 beds for patients with COVID-19. A comprehensive reorganisation of inpatient and outpatient services, development of operational and clinical guidelines with training, and establishment of a consultant-led multidisciplinary team to provide dedicated COVID-19 care was also done.

**Figure 2:**
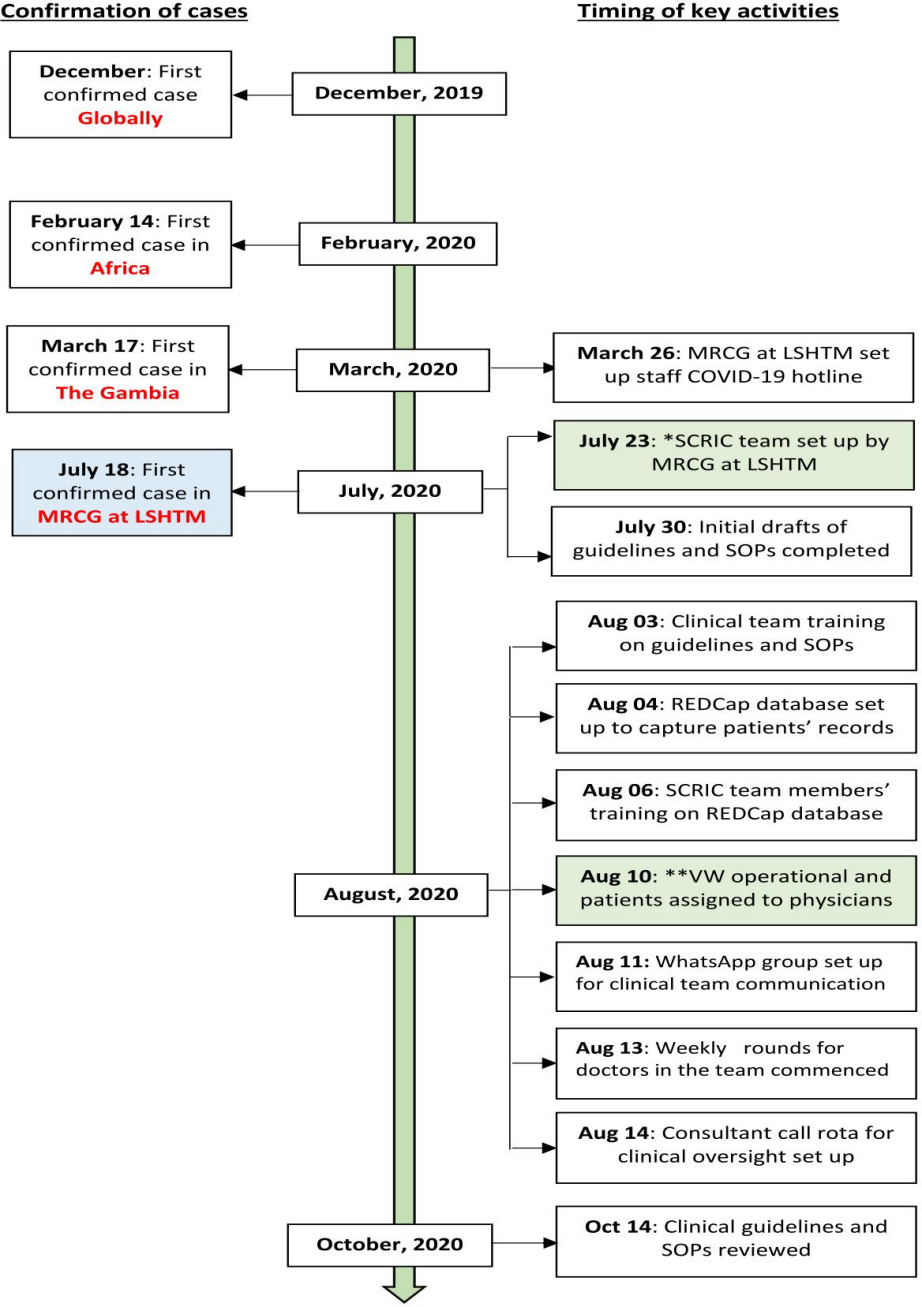
Timing of key events leading up to the establishment and operationalisation of the VW system at MRCG at LSHTM. Note: The key activities highlighted here only relates to the events preceding the setting up, and operationalisation of the SCRIC team/VW and not the general COVID-19 preparedness throughout the CSD or the MRCG at LSHTM in general. CSD, Clinical Services Department; LSHTM, London School of Hygiene and Tropical Medicine; MRCG, Medical Research Council Unit The Gambia; REDCap, Research Electronic Data Capture; SCRIC, Staff COVID-19 Risk Coordination; SOPs, standard operating procedures; VW, virtual ward.

Four months after the index case was confirmed in The Gambia, the first case of COVID-19 among an MRCG at LSHTM staff was identified on 18 July 2020 and cases subsequently increased rapidly as illustrated in figure 1. After the confirmation of the first staff case, the MRCG at LSHTM constituted a Staff COVID-19 Risk Coordination (SCRIC) team on 23 July 2020 to coordinate responses and actions and ensure the highest possible safety standards for staff. The SCRIC team commenced operation of the VW system when it became apparent that the hospitalisation of all asymptomatic and symptomatic COVID-19 cases, as was the procedure at the time, was not sustainable because it added intense pressure on the limited CSD beds and frontline HRH. See figure 2 showing the timeline of the key events leading up to the establishment and operationalisation of the VW system.

## The remit of the ‘virtual ward’ system

The objectives of the VW system is to provide home-based support for a subset of COVID-19 cases meeting certain criteria, ensure they are closely monitored and escalate if they deteriorate, thereby avoiding unnecessary hospital admission for COVID-19. The eligibility criterion for our VW system is a primary diagnosis of COVID-19 in patients who have not already been admitted to the hospital. Such patients must have asymptomatic or mild/moderate symptoms of the disease and be staff members (or their immediate relatives) who, based on their clinical presentation and assessment of risk factors by a clinician, are at low risk of progressive disease. Because our VW system requires that patients be primarily isolated at home, another eligibility requirement is that the home and social setting must be ideal for home isolation.

**Figure 3:**
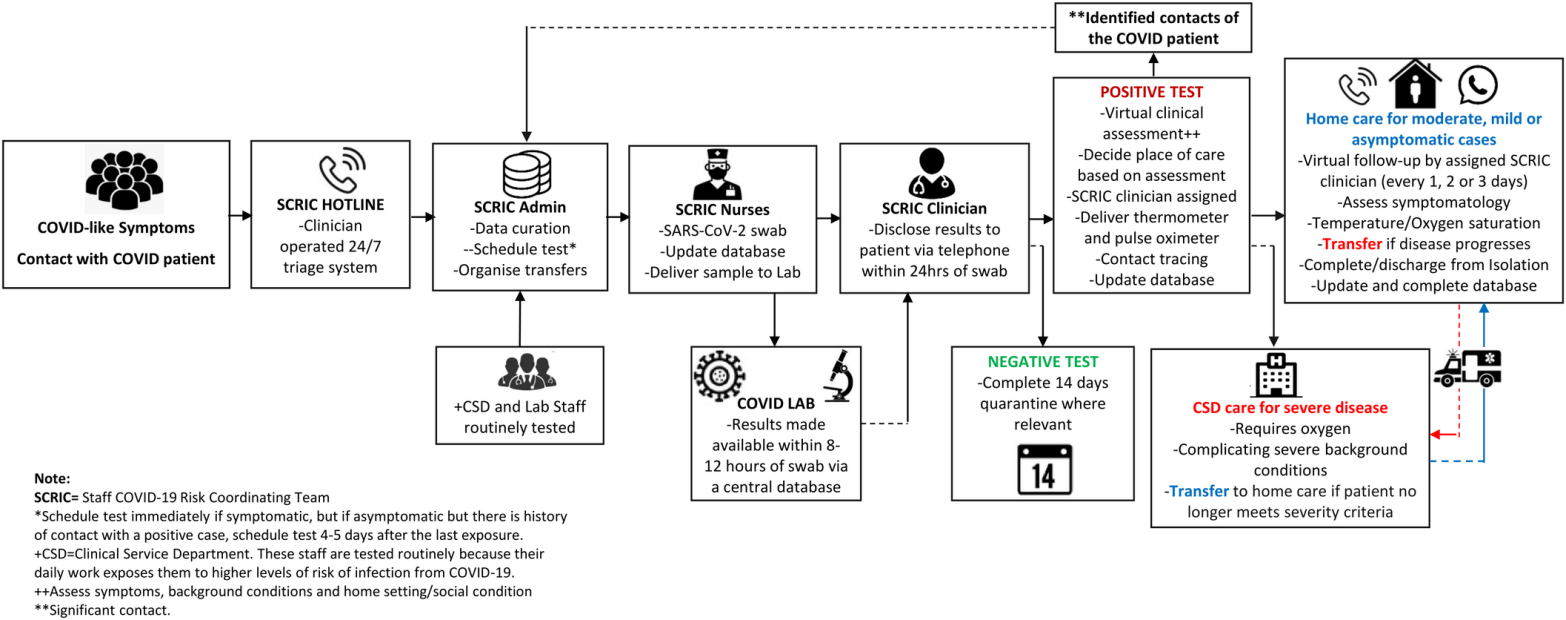
Key components of the VW system for caring for COVID-19 at home as operated at the MRCG at LSHTM. LSHTM, London School of Hygiene and Tropical Medicine; MRCG, Medical Research Council Unit The Gambia; VW, virtual ward.

The key components of our VW system are shown in figure 3. To access care through the system, staff and their immediate relatives with symptoms of COVID-19 or those who have had significant contact with a confirmed case are advised to call the staff COVID-19 hotline (ie, the internal hotline which operates 24 hours a day, 7 days a week) about their symptoms and for general enquiries. The internal COVID-19 hotline is operated by a team of clinicians who use a set of questions and a checklist adapted from the WHO guidelines to triage patients and determine who would require a diagnostic test. A SARS-CoV-2 PCR test is then organised for those who meet the requirements and results are communicated to them within the next 12–24 hours. The subsequent pathway of care (ie, virtual care or hospital admission) is determined based on the individual’s test result and severity of symptoms.

All patients confirmed COVID-19-positive and who qualify for virtual care are assigned an SCRIC physician and provided with a digital thermometer to monitor daily axillary temperature. Pulse oximeter are provided on a case-by-case basis to aid the monitoring of oxygen saturation. Pulse oximeters are given to patients who have a significant background medical condition, those who are above 55 years, or those considered by their assigned physician to have a high risk of respiratory deterioration. The assigned SCRIC physician adopts a virtual monitoring plan based on standard COVID-19 care guidelines and provides additional support as required during scheduled follow-up teleconsultation. The temperatures and oxygen saturation readings taken by the patients are fed back to physician during each ‘VW rounds’, and patients are also instructed to immediately call the SCRIC physician if their readings were above or below certain predetermined thresholds. The VW system also provides a safe accommodation on site (ie, within the MRCG at LSHTM facilities) for staff isolation where home circumstances were not suitable for home isolation. If any patient in the VW developed severe symptoms, they were reviewed and transferred to the MRCG at LSHTM CSD for inpatient hospital care.

From 24 August 2020 when the VW became operational to 31 April 2021, 427 patients who are COVID-19 positive (MRCG at LSHTM staff and their relatives) have been admitted, followed-up and discharged through the VW system. Of these, 75% (321/427) were staff, while 25% (106/427) were immediate relatives of staff (table 1). So far, about 2% of the 427 cases managed through the system developed severe disease warranting in-hospital care and were transferred to the MRCG at LSHTM CSD. So far, all the cases with severe disease have been successfully managed in the CSD, transferred back into the VW and discharged from follow-up in the VW with no mortality recorded.

**Table 1:** Demographic characteristics of 427 MRCG at LSHTM staff and relatives cared for through the VW between 24 July 2020 and 31 April 2021.

Characteristics	MRCG at LSHTM staff (N=319) n (%)	Relatives (N=108) n (%)
Age		
Range	22–63 years	4–72 year
Median	36 years	25.3 years
Race/ethnicity		
Africans	313 (98.1)	105 (97.2)
Non-Africans	6 (1.9)	3 (2.8)
Sex		
Male	175 (55)	53 (49.1)
Female	144 (45)	55 (50.9)

LSHTM, London School of Hygiene and Tropical Medicine; MRCG, Medical Research Council Unit The Gambia; VW, virtual ward.

## The composition and function of the team

To ensure the effective operation of the VW system, and provide quality COVID-19 follow-up and care, we constituted a multidisciplinary team made up of a cross-section of clinical staff (14 physicians and 10 nurses who are duly registered as practitioners in The Gambia), four project management and administrative staff, and a dedicated ambulance driver. Furthermore, we leveraged existing structures within the MRCG at LSHTM, working closely with our CSD, health and safety department, and clinical and research laboratories, which also continue to support the national SARS-CoV-2 PCR testing throughout the country.

The physicians on the team are responsible for communicating with known contacts of cases to confirm exposure, organise for such contacts to undergo a diagnostic test 3–5 days after the last known exposure, communicate positive results and provide clinical assessment and care during the VW round. The nursing staff collect the diagnostic nasopharyngeal and oropharyngeal swabs on an outpatient basis at the MRCG at LSHTM and transport samples to the clinical laboratory for SARS-CoV-2 PCR testing. The project management and administrative staff handle data entry, non-clinical communication and ensure smooth day-to-day running of the system. The ambulance drivers are on hand to provide safe transport for patients who may require transfer from their homes to the CSD for testing, or clinical evaluation if their clinical condition necessitates a transfer at any time during their follow-up.

At the beginning of the pandemic, physicians who found themselves away from The Gambia and unable to return due to travel restrictions at the time were drafted into the team if they wished to support the VW system. This was possible because of the virtual nature of our system, which enabled our physicians to provide care for patients with COVID-19 remotely. We have also been able to leverage the services of other physicians among our staff who are unable to provide face-to-face clinical services or continue with research activities because they have been assessed to be at risk for severe COVID-19 as a result of their background medical condition that warrants their shielding. Through this approach, we have been able to operate a diverse team of physicians with varied experience and located across three continents (Africa, Europe and North America). Thus, we continue to use critical clinical manpower that would have ordinarily not been available and complement the clinical care provided by the team on the ground in The Gambia as the pandemic continues.

## Clinical oversight

An assigned physician member of the team (ie, an individual who is medically qualified to provide clinical patient care and registered in The Gambia) has primary clinical oversight for each patient being followed-up through the VW system. To ensure consistency of the process and continuity of care provided, each patient had one SCRIC physician assigned to them from admission into the VW until they are discharged from follow-up. There is evidence suggesting that such an approach to follow-up helps in building trust between doctor and patient,^28^ and also allays patient’s anxiety—an outcome which is desired as the COVID-19 pandemic continues to evolve. The SCRIC clinical team operates a rota system, which ensures that one clinical consultant on the team is placed on call per week and has overall oversight for all the patients being followed-up in the VW during their period of call.

Should a patient’s clinical condition warrant additional review, it is the responsibility of the managing SCRIC physician to discuss their assigned patient with the SCRIC consultant on-call and make key clinical management decisions together. Where a patient’ symptoms progresses and warrant inpatient care, the SCRIC consultant on-call discusses the case with the CSD consultant that is responsible for providing inpatient management for severe patients with COVID-19 admitted to the CSD of the MRCG at LSHTM. A joint decision (between the SCRIC consultant and the CSD consultant) is reached before such patients are transferred for inpatient clinical care. Once patients are transferred from the VW system to the CSD for inpatient care, the CSD team takes over clinical oversight and manages the patients according to the CSD COVID-19 clinical guidelines for inpatients. Patients discharged from in-hospital care after the successful management of their severe disease at the CSD are also discussed with the SCRIC consultant on-call by the CSD consultants before being transferred to continue care in the VW system.

## Clinical standard operating procedures and guidelines

There is evidence supporting the potential positive impact of clinical guidelines and standard operating procedures (SOPs), on improving the quality and the consistency of care provided by teams of clinicians to patients.^29 30^ The SCRIC team developed SOPs and guidelines, which provides clear and stepwise processes and govern all the aspects of care that the team provides (see Box 1). These guideline documents clearly specify team members’ responsibilities and provide a detailed and stepwise description of each process. Example of issues covered by these SOPs and guidelines include how staff can access the system, how and when swabbing for SARS-CoV-2 swabbing is arranged, what clinical information to be collected before and after a test to aid informed decisions, who communicates test results and how this should be done. There are also guidelines on how the initial triage of patient is to be is conducted (online supplemental appendix 1), what criteria determines if a patient would require hospital care rather than a virtual follow-up, how and when the subsequent follow-up should be conducted and the process of terminating virtual follow-up.

10.1136/bmjgh-2021-005883.supp1 Supplementary data

Box 1 **Example of clinical guidelines and SOPs developed for the virtual ward (VW) system**

- Guideline for initial triage of patients.
- Guideline specifying how and when SARS-CoV-2 testing is arranged.
- Standard operating procedure (SOP) on the communication of SARS-CoV-2 test results to patients.
- Flowchart to aid decision on pathway of care after a positive result.
- SOP for virtual clinical assessment and follow up visits for patients who are COVID-19 positive.
- Guideline for the transfer of patients between the VW and inpatient COVID-19 care.
- Guideline for quarantine and isolation of patients.
- SOP for discharging patients with COVID-19 from the VW.

At the outset, we prioritised the drafting of these guidelines and the training of team members to ensure they were comfortable with them before their implementation. All guidelines are drafted and are continuously updated in line with the best available evidence at the time, current WHO COVID-19 guidelines and recommendations, and put into the context of the prevailing national guidelines being implemented in The Gambia.

## Teleconsultation and communication systems

To conduct virtual consultation and follow-up, we relied on mobile telephones and messaging applications such as WhatsApp. Most members of staff at the MRCG at LSHTM are connected by a closed user group on their mobile phones through a local telecommunication service provider; hence, the default mode of teleconsultation and communication between team members was the use of mobile telephones. However, ‘VW rounds’ were also conducted through WhatsApp by our team of physicians who were outside The Gambia and in scenarios where video consultation was explicitly required by the team on the ground. We resorted to the use of WhatsApp as a telecommunication tool in these scenarios because it is an inexpensive solution suitable for our physicians abroad who faced prohibitive cost of international phone. Additionally, WhatsApp video-conferencing offered a platform and the ability to conduct a more complete clinical assessment.^31^ Although internet penetration (ie, individuals using the internet) was about 20% of the general Gambian population as of January 2020,^32^ the much higher internet penetration for staff members of the MRCG at LSHTM made the use of the video-conferencing option more feasible.

## Data capture and management

To ensure a seamless virtual management of patients with COVID-19 by our team working remotely together, we use the Research Electronic Data Capture (REDCap) database to capture, share and securely store patient data. REDCap is a web-based application that provides a highly secured environment, suitable for storage of sensitive patient information and which is intuitive to use for research and clinical teams.^33^ The database was designed by the MRCG at LSHTM data management team, in collaboration with the SCRIC team and all the clinical team members have remote access to the database. Patient data captured in the database include basic biodata, history of contact/exposure to confirmed cases, symptomatology, background medical conditions and results of all SARS-Cov-2 tests conducted. We also capture all clinical notes and decisions made throughout the virtual follow-up process.

## Limitations and challenges of the virtual ward system

The implementation and sustainability of our VW system has been subject to two main challenges. The first has been maintaining optimal staffing to operate the VW system. At the time the system was implemented at the peak of the first wave of the pandemic in The Gambia in mid-2020, most non-essential activities in our unit had been halted thereby enabling secondment of some of the clinical and non-clinical staff from their primary research, and administrative roles to support the system. With the resumption of normal duties after the first wave, many staff have returned to their routine duties. Although they still continue to provide some support, the demands of their routine workloads limits how much support they can reasonably provide. The second challenge has been securing continued funding to sustain the additional staffing, consumables and logistics requirement for the system. Financial resources are finite, even more so during a pandemic, which continue to stretch to the limits existing budget lines. An economic analyses of the VW will be useful to highlight the utility and potential cost savings, or the additional cost of operating the VW system.

## Potential implementation and operational challenges for other settings

For the successful establishment and operation of the VW in other resource-limited settings, there are potential implementation bottlenecks that must be considered. First, internet penetration in most resource-limited setting is sometimes patchy and could impede the success of the VW system, especially for video-conferencing and electronic data capture. How poor internet penetration and low quality bandwidth hampers the feasibility and utility of remote healthcare delivery models in sub-Saharan is well documented.^15^ We overcame this operational challenge because internet penetration is generally high among the cohort of people the system supported and the MRCG at LSHTM has an excellent network bandwidth hosting the electronic data capture system, thus, making data capture seamless. Although many sub-Saharan Africa settings lack optimal internet and digital infrastructure, the use of the paper-based diaries and phone call VW models could bridge this gap even as the pandemic evolve across Africa.

Second, although this VW system has worked well so far in our setting, as mentioned above, it is not without cost to the MRCG at LSHTM. While many VW systems are embedded within existing healthcare budgets,^22^ ensuring their long-term sustainability requires additional funding. Thus, detailed budgeting for additional cost such as data capture systems, telephone communication, internet charges, consumables (thermometers, pulse oximeters, etc) and logistics must be considered before the implementation of the VW system. This is even more important especially in sub-Saharan Africa, where such cost is often not equitably budget for at primary-level and secondary-level public healthcare facilities.^34^

Third, the VW requires HRH who can dedicate time to run the system. Similar to the model we operated, during the COVID-19 pandemic, many VW systems in high-income countries reallocated HRH from non-emergency clinical setting to support the implementation of the model.^35 36^ Nevertheless, HRH in many low-income and middle-income countries is limited and they are already overstretched from supporting in-hospital care for COVID-19 and other diseases, thus, potentially limiting their availability to support a VW system. The feasibility of implementing VWs in sub-Saharan Africa requires that careful attention be paid to the potential implementation challenges we have highlighted above. Ultimately, even in high-income countries, the sustainability of VW systems to provide care for patients with COVID-19 in the future will depend on how these challenges are navigated.

## Conclusion

We have described the processes involved in setting up and operating a successful VW system at the MRCG at LSHTM to manage staff and their family members who developed asymptomatic, mild or moderate COVID-19. We demonstrate that a VW system for the care of patients with COVID-19 in non-hospital setting is feasible in a low-income country in west Africa. We believe that other low-income or resource-constrained settings can adopt and contextualise the processes described here to safely support patients with COVID-19 in non-hospital settings.

## Data Availability

There are no data in this work.

## References

[1] Worldometer. Coronavirus update: 119,895,303 cases and 1,989,457 deaths from COVID-19 virus pandemic, 2021.

[2] WHO Regional Office for Africa. COVID-19 cases top 10 000 in Africa 2020. Available: https://www.afro.who.int/news/covid-19-cases-top-10-000-africa#:~:text=Reaching%20the%20continent%20through%20travellers,countries%20have%20reported%20cases

[3] Burki T. The indirect impact of COVID-19 on women. Lancet Infect Dis 2020;20:904–5. 10.1016/S1473-3099(20)30568-5 32738239 PMC7836874

[4] Njenga MK, Dawa J, Nanyingi M, et al. Why is there low morbidity and mortality of COVID-19 in Africa? Am J Trop Med Hyg 2020;103:564–9. 10.4269/ajtmh.20-0474 32484156 PMC7410455

[5] Banerjee A, Pasea L, Harris S, et al. Estimating excess 1-year mortality associated with the COVID-19 pandemic according to underlying conditions and age: a population-based cohort study. Lancet 2020;395:1715–25. 10.1016/S0140-6736(20)30854-0 32405103 PMC7217641

[6] World Economic F. Why Sub-Saharan Africa needs a unique response to COVID-19, 2020.

[7] Martinez-Alvarez M, Jarde A, Usuf E, et al. COVID-19 pandemic in West Africa. Lancet Glob Health 2020;8:e631–2. 10.1016/S2214-109X(20)30123-6 32246918 PMC7186549

[8] Gilbert M, Pullano G, Pinotti F, et al. Preparedness and vulnerability of African countries against importations of COVID-19: a modelling study. Lancet 2020;395:871–7. 10.1016/S0140-6736(20)30411-6 32087820 PMC7159277

[9] Lone SA, Ahmad A. COVID-19 pandemic - an African perspective. Emerg Microbes Infect 2020;9:1300–8. 10.1080/22221751.2020.1775132 32458760 PMC7473237

[10] Chersich MF, Gray G, Fairlie L, et al. COVID-19 in Africa: care and protection for frontline healthcare workers. Global Health 2020;16:46. 10.1186/s12992-020-00574-3 32414379 PMC7227172

[11] Freer J. Sustainable development goals and the human resources crisis. Int Health 2017;9:1–2. 10.1093/inthealth/ihw042 27815421

[12] Mehtar S, Preiser W, Lakhe NA, et al. Limiting the spread of COVID-19 in Africa: one size mitigation strategies do not fit all countries. Lancet Glob Health 2020;8:e881–3. 10.1016/S2214-109X(20)30212-6 32530422 PMC7195296

[13] Word Health Organization. Digital health and COVID-19. WHO, 2020.

[14] Lewis C, Moore Z, Doyle F, et al. A community virtual ward model to support older persons with complex health care and social care needs. Clin Interv Aging 2017;12:985–93. 10.2147/CIA.S130876 28721026 PMC5498784

[15] Babalola D, Anayo M, Itoya DA. Telehealth during COVID-19: why sub-Saharan Africa is yet to log-in to virtual healthcare? AIMS Medical Science 2021;8:46–55. 10.3934/medsci.2021006

[16] Thornton J. The "virtual wards" supporting patients with covid-19 in the community. BMJ 2020;369. 10.1136/bmj.m2119 32499317

[17] Annis T, Pleasants S, Hultman G, et al. Rapid implementation of a COVID-19 remote patient monitoring program. J Am Med Inform Assoc 2020;27:1326–30. 10.1093/jamia/ocaa097 32392280 PMC7239139

[18] Schultz K, Vickery H, Campbell K, et al. Implementation of a virtual ward as a response to the COVID-19 pandemic. Aust Health Rev 2021. 10.1071/AH20240. [Epub ahead of print: 12 Apr 2021]. 33840420

[19] Ferry OR, Moloney EC, Spratt OT, et al. A virtual ward model of care for patients with COVID-19: retrospective single-center clinical study. J Med Internet Res 2021;23:e25518. 10.2196/25518 33529157 PMC7879714

[20] Shah S, Majmudar K, Stein A, et al. Novel use of home pulse oximetry monitoring in COVID-19 patients discharged from the emergency department identifies need for hospitalization. Acad Emerg Med 2020;27:681–92. 10.1111/acem.14053 32779828 PMC7323027

[21] Vindrola-Padros C, Singh KE, Sidhu MS. Remote home monitoring (virtual wards) during the COVID-19 pandemic: a systematic review. medRxiv 2021. 10.1016/j.eclinm.2021.100965 PMC8219406 34179736

[22] Vindrola-Padros C, Sidhu MS, Georghiou T, et al. The implementation of remote home monitoring models during the COVID-19 pandemic in England. EClinicalMedicine 2021;34:100799. 10.1016/j.eclinm.2021.100799 33817610 PMC8008987

[23] The World Bank. Current health expenditure per capita (current US$) - Gambia, The 2021. Available: https://data.worldbank.org/indicator/SH.XPD.CHEX.PC.CD?locations=GM

[24] UN Department of Economic and Social Affairs Population Dynamics. World population prospects 2019 2019. Available: https://population.un.org/wpp/Download/Standard/Population/

[25] World Health Organization. Global Health Observatory: Gambia - statistics summary (2002 - present)

[26] Ministry of Health The Republic of The Gambia. Guidelines for quarantine, isolation and testing. guideline. Banjul, the Gambia: Ministry of health the Gambia, 2020 [Accessed Aug 10, 2020].

[27] World Health Organization. Criteria for releasing COVID-19 patients from isolation 2020.

[28] Chipidza FE, Wallwork RS, Stern TA. Impact of the doctor-patient relationship. Prim Care Companion CNS Disord 2015;17. 10.4088/PCC.15f01840. [Epub ahead of print: 22 10 2015]. PMC4732308 26835164

[29] Woolf SH, Grol R, Hutchinson A, et al. Clinical guidelines: potential benefits, limitations, and harms of clinical guidelines. BMJ 1999;318:527–30. 10.1136/bmj.318.7182.527 10024268 PMC1114973

[30] Johnston A, Kelly SE, Hsieh S-C, et al. Systematic reviews of clinical practice guidelines: a methodological guide. J Clin Epidemiol 2019;108:64–76. 10.1016/j.jclinepi.2018.11.030 30529647

[31] De Benedictis A, Lettieri E, Masella C, et al. WhatsApp in hospital? An empirical investigation of individual and organizational determinants to use. PLoS One 2019;14:e0209873–e. 10.1371/journal.pone.0209873 30633754 PMC6329505

[32] The World B. Individuals using the Internet (% of population) - Gambia, The.. Available: https://data.worldbank.org/indicator/IT.NET.USER.ZS?locations=GM [Accessed Feb 10, 2021].

[33] Patridge EF, Bardyn TP. Research electronic data capture (REDCap). J Med Libr Assoc 2018;106:42–4. 10.5195/JMLA.2018.319

[34] Goodyear-Smith F, Bazemore A, Coffman M, et al. Primary care financing: a systematic assessment of research priorities in low- and middle-income countries. BMJ Glob Health 2019;4:e001483. 10.1136/bmjgh-2019-001483 PMC6703294 31478025

[35] et alMorgan A, Balachandran M, Do D. Remote monitoring of patients with COVID-19: design, implementation and outcomes of the first 3,000 patients in COVID Watch. Available: https://catalyst.nejm.org/doi/full/10.1056/CAT.20.0342 [Accessed May 20, 2021].

[36] Misra-Hebert AD, Ji X, Jehi L, et al. COVID-19 home monitoring after diagnosis and health care utilization in an integrated health system. JAMA Health Forum 2021;2:e210333. 10.1001/jamahealthforum.2021.0333 PMC8796892 35977306

