# Supplementary material for: Establishing and operating a ‘virtual ward’ system to provide care for patients with COVID-19 at home: experience from The Gambia
Source: BMJ Glob Health. 2021 Jun 17;6(6):e005883. doi: 10.1136/bmjgh-2021-005883 (PMC8212157; doi:10.1136/bmjgh-2021-005883)
Supplement: Supplementary data [file bmjgh-2021-005883supp001.pdf]

MRC Unit The Gambia   LONDON SCHOOL of HYGIENE &TROPICAL MEDICINE

CHECKLIST FOR INITIAL TRIAGE AND REVIEW OF STAFF AND THEIR RELATIVE

WHO TESTED POSITIVE FOR COVID-19

Disclosure of positive results to staff and their relatives should be done confidentially and with empathy considering the effect such news may have on the person.

INFORMATION TO BE OBTAINED

1 **History of background/ underlying disease/ immunocompromised state**.

2 Presence of **ongoing symptoms** and history of symptoms in the past. (History of symptoms with date of onset, severity of symptoms especially respiratory symptoms and the diurnal pattern of body temperature/fever).

a) If symptoms are ongoing; self-isolation period will be for 13days; if after 13 days symptoms still persist or new symptoms develop within the isolation period (fever, respiratory symptoms), the period is extended until the staff or relative is symptom free for 3 days (see appendix 1). For a staff who previously had symptoms, but on the day of result disclosure is currently asymptomatic, rule (2b) applies

b) For asymptomatic staff/relative who do not develop any symptom in the course of self-isolation, the period of self-isolation is for 10days

c) For the "significant contacts" of the staff or relative, they proceed for quarantine for 14 days (see appendix 1), and if any of them develop symptoms they are tested,

a. if ***positive*** they will be treated as in case (2a),

b. if ***negative*** they complete the 14 days self-isolation.

3 **Home setting/ social factors**

a) Number of rooms in the house

b) Number of persons who live in the house (under the same roof)

c) Room occupancy pattern (the number of people sleeping in a room, distribution of rooms within the family members.

d) Number of convenience present and the number of persons who use the convenience

e) Activities done in common in the house (Eating together, playing games, amongst others)

f) Number of persons in the compound and the interactions between them (the activities they do in common).

g) Anyone in the compound with COVID-relevant underlying conditions?

INFORMATION DISCLOSED TO COVID-19 POSITIVE STAFF AND RELATIVES

1 The Result (*Positive, Indeterminate*, Negative*).

2 The meaning of the result and ensure feedback is gotten from the staff or relative that they understand the information just given.

3 Based on information obtained, *asymptomatic*, *mild*, and *moderate* cases of COVID-19 are told to proceed on self-isolation.

Staff COVID-19 Risk Coordination Team SCRIC

4 The expected duration of self-isolation for the staff/ relative and the contacts in the house is communicated.

5 During this time, MRC clinicians from the SCRIC team will keep in touch with staff and their relatives to monitor their condition and ensure self-isolation at home continues to be appropriate; and in case of deterioration an admission to CSD or a treatment Centre will be considered.

6 Rules of self-isolation are explained to them

a) Sleeping alone in a room and spending as much time as possible alone in this room
b) Not leaving the compound during the self-isolation period
c) Use of facemask in the house or when talking to others
d) Regular washing of hands with soap and water especially after touching commonly used surfaces like door handles etc.
e) Disinfecting frequently touched surfaces and the floor with sodium hypochlorite (bleach) in water
f) Maintaining at least "2m" from contacts in the home and within the compound
g) Exposure to early morning sun within the compound
h) Eating a balanced diet
i) Adequate aeration and ventilation of the rooms in the house
j) Proper hydration
k) Rest
l) The need to communicate to the assigned follow-up doctors if a new symptom develops or a "Significant contact" develops a symptom and requires testing
m) Taking of paracetamol as antipyretics, and analgesic if the need arises.

7 Name of Project/Department and contact details of their line manager (name, email)- only applicable to staff. Note that staff should be told that their line managers would be informed about their status or that of their relative if it affects their mobility for purposes of contact tracing and so that they can be excused from duty for the duration of isolation.

8 Staff is told to inform their line manager

Note:** for ***indeterminate result, the staff/ relative is asked to re-swabbed 2 days later.

ACTIONS TO BE TAKEN AFTER SPEAKING WITH THE STAFF OR RELATIVE

1 **Send an email to the line manager** of the affected staff and copy in HR. This does not apply to patient relatives per se, but only if it affects the staff in their mobility due to isolation requirements.

2 Complete the section on REDCap and check the box which indicates that the line manager has been informed

3 **Assign the staff or their relative** to a specific SCRIC physician by adding their names to the page of the assigned physician on the live SCRIC daily follow-up excel sheet

4 If a staff or their relative needs to isolate at the MRC, the nurse coordinator on the SCRIC team (Mr Bunja-2954157) should be informed to make this arrangement.

Staff COVID-19 Risk Coordination Team SCRIC

5 If a staff or their relative needs to be admitted to the CSD, discuss with the SCRIC consultant on call for the week and agree on the decision before reaching out to the CSD COVID consultant on call. Names and numbers of the CSD COVID consultant on call shall be provided weekly to the SCRIC team.

**Written by:**	Name: Emmanuel Okoh	Date: 23 July 2020
**Reviewed by:**	Name: Oghenebrume Wariri	Date: 26 July 2020
**Reviewed by:**	Name: Hawanatu Jah	Date: 26 July 2020
**Version:**	**Change history:**	**Review due date:**
1.0	New document	26 July 2022
Review Comments (*if applicable)*		
